# Influence of Cross-Linking Agent Concentration/Beta Radiation Surface Modification on the Micro-Mechanical Properties of Polyamide 6

**DOI:** 10.3390/ma14216407

**Published:** 2021-10-26

**Authors:** Martin Ovsik, Michal Stanek, Adam Dockal, Jiri Vanek, Lenka Hylova

**Affiliations:** Faculty of Technology, Tomas Bata University in Zlin, Vavreckova 275, 760 01 Zlín, Czech Republic; stanek@utb.cz (M.S.); a_dockal@utb.cz (A.D.); j4_vanek@utb.cz (J.V.); hylova@utb.cz (L.H.)

**Keywords:** polyamide, triallyl isocyanurate, electron beam, micro-indentation, indentation hardness, Fourier-transform infrared spectroscopy

## Abstract

This study focuses on the problematic of polyamide 6 containing various concentrations of cross-linking agent that was exposed to electron radiation. It is important to improve the material properties of polymers as much as possible. This endeavor can be realized by numerous methods, one of which is radiation exposure. This study investigates the effect of electron beam radiation in doses ranging from 66 to 132 kGy on the micro-mechanical properties of polymers, specifically polyamide 6 filled with 1, 3 and 5 wt.% of cross-linking agent triallyl isocyanurate (TAIC). The changes in the material brought by the radiation exposure were quantified by measurements of indentation hardness and modulus, which were the main measured micro-mechanical properties. Furthermore, thermo-mechanical analysis (TMA) was chosen to confirm the results of the material cross-linking, while the effect of degradation was investigated by Fourier-transform infrared spectroscopy (FTIR). In pursuit of complete evaluation, the topography of the test subject’s surface was explored by atomic force microscopy (AFM). The optimal concentration/radiation ratio was found in polyamide 6 enriched by 5 wt.% concentration of TAIC, which was irradiated by 132 kGy. Material treated in such a way had its indentation hardness by 33% and indentation modulus improved by 26% in comparison with the untreated material. These results were subsequently confirmed by the TMA and FTIR methods.

## 1. Introduction

Cross-linking of polymers is a process, which leads to the connection of macro-molecular chains in a 3D spatial network. Cross-linking of polymer materials induced by beta radiation is done by accelerated electrons that are produced by an electron accelerator. Beta radiation is commonly used for most plastic components, since this treatment of polymer materials requires a relatively high dose of radiation. Fortunately, a high energy output of generally used electron accelerators enables quick manufacturing speeds. Additionally, parts are usually irradiated from both sides, which improves the economic aspect of the process.

The cross-linking of polymers is a chemical process, during which transverse bonds are created within the polymer structure. Due to these bonds, an infinite 3D structure, i.e., spatial network or gel, is created. Radiation-induced cross-linking is caused by the collision of moving free radicals, especially in amorphous regions of polymers.

The cross-linking process usually consists of two contradictory events happening at the same time, covalent bond formation and scission (the decomposition of the main chain-link). These processes have the following effects on polymers: increase of molecular weight; lower solubility in organic solvents; and improved chemical, thermal and mechanical properties.

The degree of cross-linking depends on numerous factors, e.g., crystallinity, temperature of glass transition, chemical structure. In order to reach the best possible result, it is important to carefully consider these factors [1].

Another important factor in the realm of polymer cross-linking is the amount of absorbed radiation dose, which is a function of radiation unit’s exit velocity in a given polymer structure. This function makes cross-linking a reliable process, as is it possible to regulate all the crucial parameters [1].

The effect of high-energy radiation on polymer materials’ properties is complex and dependent on polymer structure, molecular weight, polymer status, and the degree of crystallinity. The speed of the irradiation and the ambient atmosphere during the process are also main factors. Moreover, covalent bond formation, scission and creation of gas are the main processes. These processes lead to changes in the polymer properties [1,2].

Polyamides belong to a group of polymers, which can be cross-linked by both the electron beam and gamma radiation technology. In addition, this polymer displays covalent bond formation, as well as the scission process during the irradiation. The gain in mechanical properties corresponds with the amount of methyl groups (-CH_2_-), which are present in the polyamide structure. Furthermore, higher concentrations of water absorbed in polyamide improve the cross-linking process, whereas lower concentrations of water dampen it [2].

Polyamides usually change color when exposed to either electron beam or gamma radiation. This change is caused by the creation of radicals, which commonly occurs on an α carbon neighboring an amide nitrogen. If a hydrogen atom on the α carbon is blocked, for example by a phenyl group, the gains from both cross-linking and scission are reduced [1,2].

**Figure A1:**
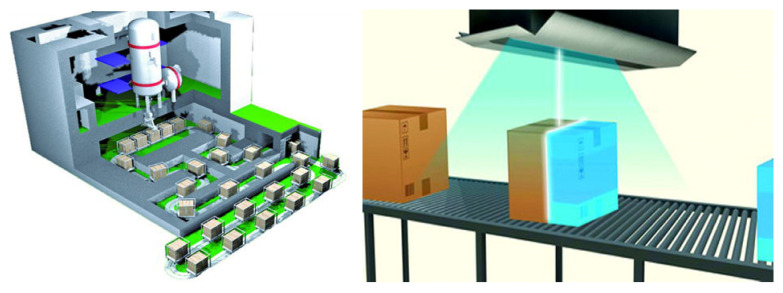
Source of electron radiation–the electron accelerator.

**Figure A2:**
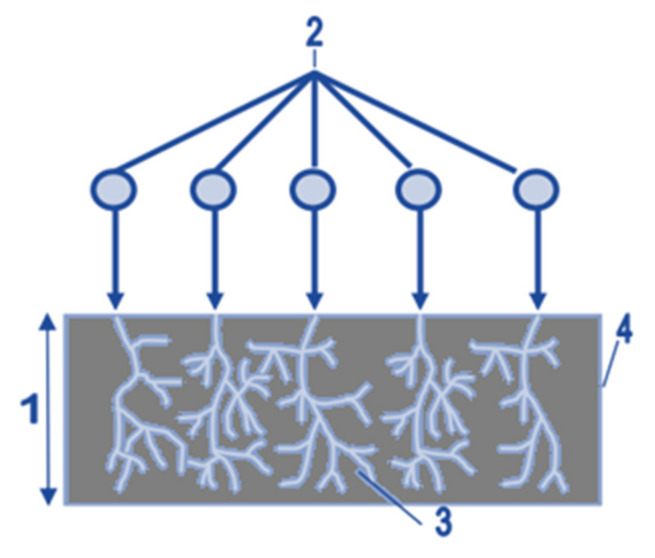
Principle of electron irradiation: 1—electron penetration depth, 2—primary electrons, 3—secondary electrons, 4—irradiated material.

As is common for many polymers, the effect of radiation on the physical properties of polyamides is heavily influenced by ambient atmosphere during the process (Figure A1). Additionally, aromatic polyamides provide higher resistance against radiation in comparison to their aliphatic counterparts (Figure A2) [1,2].

In 2005, Dadbin et al. investigated the electron radiation-induced cross-linking of PA 6, which was enriched by 1–3 wt.% of cross-linking agent triallyl cyanurate (TAC). The electron accelerator in question provided 5 MeV of energy, which enabled irradiation by various doses in a range from 40 to 150 kGy. It was found that the molecular weight of polymer samples increased with rising radiation exposure, which was subsequently confirmed by viscosity measurements. Furthermore, the gel-content test showed that PA 6 with TAC could effectively cross-link even when exposed to a lower radiation dose. The content of absorbed water declined with increasing amounts of TAC and absorbed radiation dose. [3]

In 2009, Pramanik et al. investigated the cross-linking of PA 6 with TAC that was exposed to radiation doses of 100, 200, 300, 400, 500, and 600 kGy at room temperature. The irradiation process was done by accelerated electrons with 2 MeV of energy. The hardness, strength, and flexible and impact properties of PA 6 displayed significant improvements after the process. The optimal radiation dose that led to the best improvement in the aforementioned properties was found in specimen irradiated by 400 kGy. The amounts of absorbed water declined with increasing radiation dose [4].

In 2011, Holik et al. focused on the effect of cross-linking agent concentration on the properties of irradiated polyamide 6. In order to cross-link the PA 6, accelerated electrons with 10 MeV energy and radiation doses of 66, 99, and 132 kGy were used. The specimen containing 5% of cross-linking agent irradiated by 132 kGy of radiation showed the best material properties. [5]

In the following year, scientists from the same institution studied the change of micro-mechanical properties in specimens exposed to ionizing beta radiation. PA 6 was enriched by 6 vol.% of cross-linking agent TAIC and subsequently irradiated by 0, 15, 30, 45, 66 and 99 kGy. The results of this research showed that the micro-mechanical surface properties of PA 6 significantly improved in comparison with the untreated sample. For example, micro-hardness rose by 41%, while micro-toughness increased by 50%. On the other hand, micro-creep properties declined by 16%. A radiation dose of 30 kGy was designated as the most optimal. Moreover, higher radiation doses led to a decrease of micro-mechanical properties [6].

Porubska et al., who specializes in the radiation treatment of polymer materials by various sources, studied the effect of radiation on PA 6 with 30 wt.% of glass fibers and its un-filled version in 2014. The specimens prepared by injection-molding technology were later irradiated by accelerated electrons with 10 MeV energy and radiation doses of 50, 100, 200, 300, and 500 kGy. Both sides of the samples were exposed to 50% of the radiation. The results showed that radiation treatment provided better improvements in unfilled PA 6 [7].

Adem et al. irradiated 0.6 mm thin PA 6 and PA 66 polymer films by ionizing beta radiation with 1.3 MeV energy and radiation doses in a range of 50 to 1000 kGy and in ambient temperature range from room temperature to 70 °C. It was found that stress at break and elongation at break were significantly reduced, while Young’s modulus and slip stress increased with rising radiation dose. The gel-content test showed that the cross-linked volume was greater for PA 6 than for PA 66. The melt and crystallization temperature declined with increasing radiation dose, which was caused by scission and covalent bond formation at the boundary of crystals in the amorphous part of the polymer [8].

In 2015, Manas et al. studied the influence of cross-linking agent concentration on the micro-mechanical properties of PA 6 filled with 30% of glass fibers. The test samples were prepared by injection molding. The various amounts of cross-linking agent TAIC were 0, 1, 2, and 3 vol.%. The usage of the aforementioned cross-linking agent proved to have differing results on micro-mechanical properties. While the indentation hardness along with the indentation elastic modulus declined with increasing amounts of TAIC, the indentation creep demonstrated an opposite trend. These results were caused by the presence of TAIC monomer within the blend, which decreased the micro-mechanical properties prior to the irradiation process [9].

In 2016, Shin et al. investigated the influence of electron radiation on the morphology, rheology, and mechanical properties of a polymer blend that consisted of PA 6 and PP. It was found that electron radiation improved the compatibility of the boundary between PA 6 and PP [10].

The influence of electron radiation on the mechanical and thermal properties of selected types of polymers was researched in 2018. The findings showed that, although different polymers had varying reaction to the irradiation process, improved material properties were gained when the optimal radiation dose was found [11].

Bradler et al. tested commercially available polyamides (unfilled and filled with 30 to 35 wt.% of glass fibers), which were enriched by 5 wt.% of TAIC and subsequently cross-linked by electron radiation with doses of 50, 100, 150, and 200 kGy. Following the radiation treatment, the growth of fatigue-induced tears in a temperature range of 23 to 80 °C was examined. There was no observable effect for unfilled PA 66 in an ambient temperature of 23 °C. On the other hand, the growth rate of fatigue-induced tears was worse for an ambient temperature of 80 °C while the specimen was saturated with water. Filled polyamides were measured to have the opposite trend [12].

Polyfunctional monomers containing more than two C=C bonds are commonly used to achieve improved cross-linking. These compounds are called cross-linking accelerators. Many of these accelerators, e.g., diacrylate, dimethacrylate, triacrylate, trimethacrylate, were developed to cross-link polymers with the aid of peroxide and for the thermal or UV/EB treatment of oligomers [13,14].

**Figure 1:**
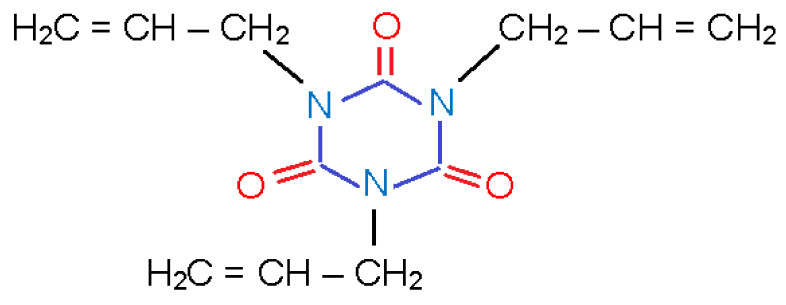
Structural formula of cross-linking accelerator TAIC.

However, highly reactive polyfunctional monomers are susceptible to polymerization by stirring or shaping under high temperature, which leads to the loss of their ability to accelerate cross-linking. Polyfunctional monomer TAIC possesses higher heat resistance than acrylate and methacrylate cross-linking accelerators (Figure 1) [14,15].

The goal of this research was the study of PA 6 irradiated by accelerated electrons with doses of 0, 66, 99, and 132 of kGy. TAIC in the following concentrations: 1, 3, and 5 wt.%, was used as the cross-linking agent. The main interest was discovering the optimal concentration of TAIC and the radiation dose, which would lead to the biggest overall improvement in micro-mechanical properties. The results of the aforementioned properties were subsequently confirmed by thermo-mechanical analysis (TMA), while proof of degradation was provided by Fourier-transform infrared spectroscopy (FTIR). Complimentary to this research, the topography of the surface was studied by atomic force microscopy (AFM).

## 2. Materials and Methods

The experimental part contains the conditions of the measurement for the micro-mechanical properties’ tests (indentation hardness and modulus), DMA, FTIR, and AFM.

### 2.1. Material

Polyamide 6 with the trade name PA 6 FRIANYL B63 VN provided by German company Frisseta (Utzenfeld, Germany) was used as the tested material. The blends with 1, 3, and 5 wt.% of cross-linking agent TAIC were prepared in cooperation with BGS Beta Gamma Service GmbH & Co, KG. Throughout this research, specimens will be called according to Table 1. In order to present well-distinguishable results, individual samples with varying amounts of cross-linking agent TAIC were designated as specimens 0, A, B, and C. The virgin material was code-named sample 0; PA 6 containing 1 wt.% of TAIC was designated as specimen A; samples with 3 wt.% of TAIC were called specimen B, and finally polyamide 6 with 5 wt.% of cross-linking agent was given the moniker of sample C.

### 2.2. Samples Preparation

The test samples with the following dimensions: (4 × 80 × 10) mm, were prepared according to ČSN EN ISO 179-1 standard with an injection-molding machine, Arburg Allrounder 470 H, constructed by the German company Arburg (Loßburg, Germany) [16,17,18]. Table 2 shows the processing conditions that were used for specimen creation.

The tested material was dried in accordance with parameters found in a material sheet (drying temperature of 120 °C, drying duration of 4 h). The required conditions were given by the granulate supplier. The drying process was conducted on THERMOLIFT 100-2 manufactured by ARBURG (Losburg, Germany). Granulate was delivered into the injection-molding machine by pneumatic suction.

### 2.3. Sample Irradiation

The specimens were sent to a German company, BGS Beta Gamma Service GmbH & Co, KG, residing in Saal an der Donau, where they were exposed to electron radiation with 10 MeV energy. One pass under the electron source exposed the material to 33 kGy. The specimens were irradiated by 0, 66, 99, and 132 kGy at room temperature and air atmosphere.

### 2.4. Micro-Indentation Properties

Micro-mechanical properties were measured on a Micro Combi Tester provided by the Swiss company CSM Instruments (Graz, Austria). The measurements were done according to CSN EN ISO 14577-1 standard. In the present study, the maximum load used was 1 N, and the loading rate (and unloading rate) was 2 N/min. The holding time was 90 s. Every specimen was fixed to metal holders, and each value was measured nine times (3 measurements on each sample) for each radiation dose and cross-linking agent concentration.

**Figure 2:**
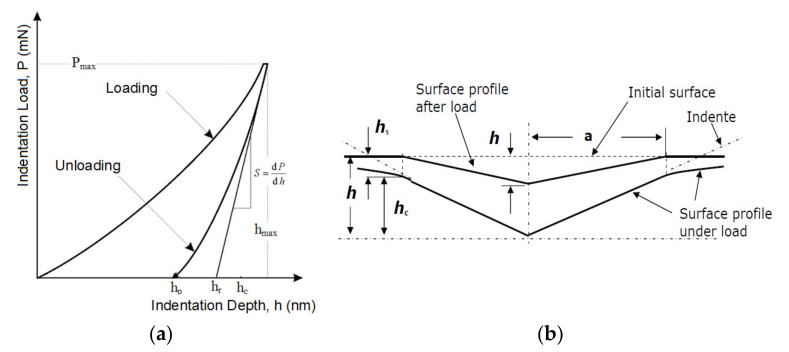
(**a**) Load–displacement curve, showing the values used in the Oliver and Pharr method; (**b**) cross-section of an indentation.

Indentation hardness (H_IT_, Figure 2) was calculated as the maximum load (*F_max_*) on the projected area of the hardness impression (*A_p_*) [19,20].

(1) $$H_{IT}=\frac{F_{max}}{A_{p}}$$

(2) $$A_{p}=23.96\cdot h_{c}^{2}$$

The indentation modulus (*E_IT_*) was calculated from the plane strain modulus (E*) using an estimated Poisson’s ratio (*ν_s_*) of the sample (Polymer 0.3 to 0.4) [20,21,22].

(3) $$E_{IT}=E^{*}\cdot ({1-v_{s}}^{2})$$

(4) $$E^{*}=\frac{1}{\frac{1}{E_{r}}-\frac{1-v_{i}^{2}}{E_{i}}}$$

(5) $$E_{r}=\frac{\sqrt{\pi }}{2\cdot C\sqrt{A_{p}}}$$

where *E_i_* is the elastic modulus of the indenter (diamond 1141 GPa); *E_r_* is the reduced modulus of the indentation contact, and *ν_i_* is the Poisson’s ratio of the indenter (0.07).

### 2.5. Thermo-Mechanical Analysis (TMA)

**Figure 3:**
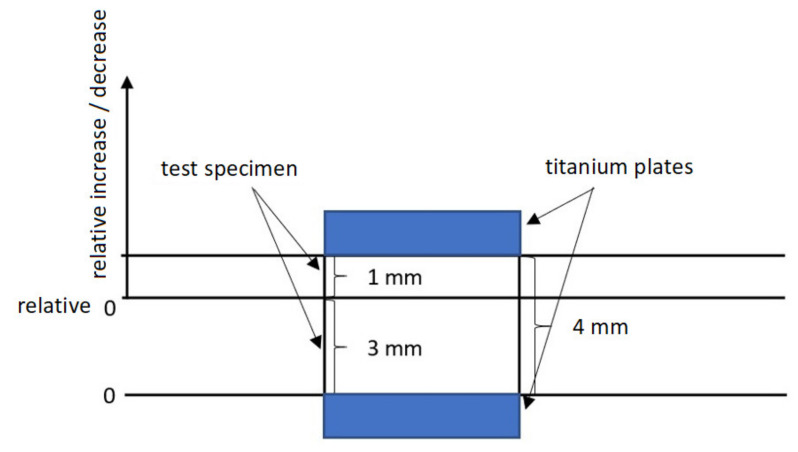
TMA measurement principle.

**Figure A3:**
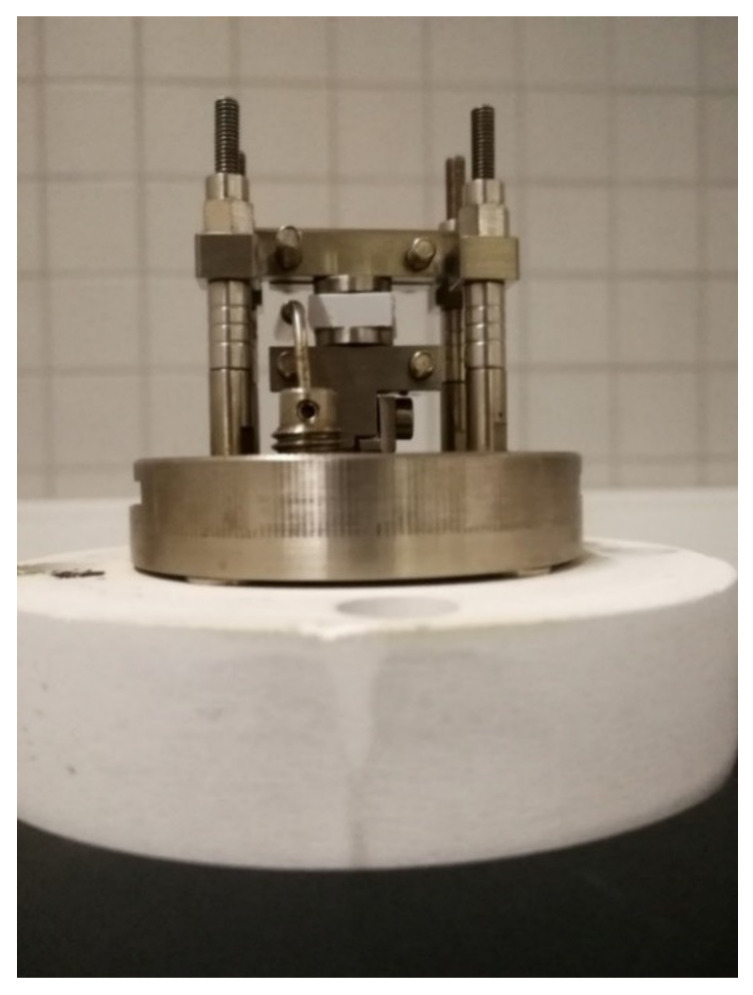
Clamping of the specimen.

The investigation of specimen’s deformation was done by a DMA machine (Figure 3 and Figure A3) constructed by METTLES TOLEDO (Langacher Greifensee, Switzerland). The conditions of the thermo-mechanical analysis of creep can be seen in Table 3.

The measurements were done on 3 test subjects with the following dimensions (10 × 4 × 10) mm for each radiation dose and TAIC concentration. The equation for deformation calculation is:

(6) $$\frac{\mathrm{deformation}\;\mathrm{of}\;\mathrm{the}\;\mathrm{specimen}\;\left(µm\right)}{\mathrm{thickness}\;\mathrm{of}\;\mathrm{the}\;\mathrm{specimen}\;\left(µm\right)}\times 100=\mathrm{deformation}\;\left(\%\right)$$

### 2.6. Fourier-Transform Infrared Spectroscopy (FTIR)

**Figure A4:**
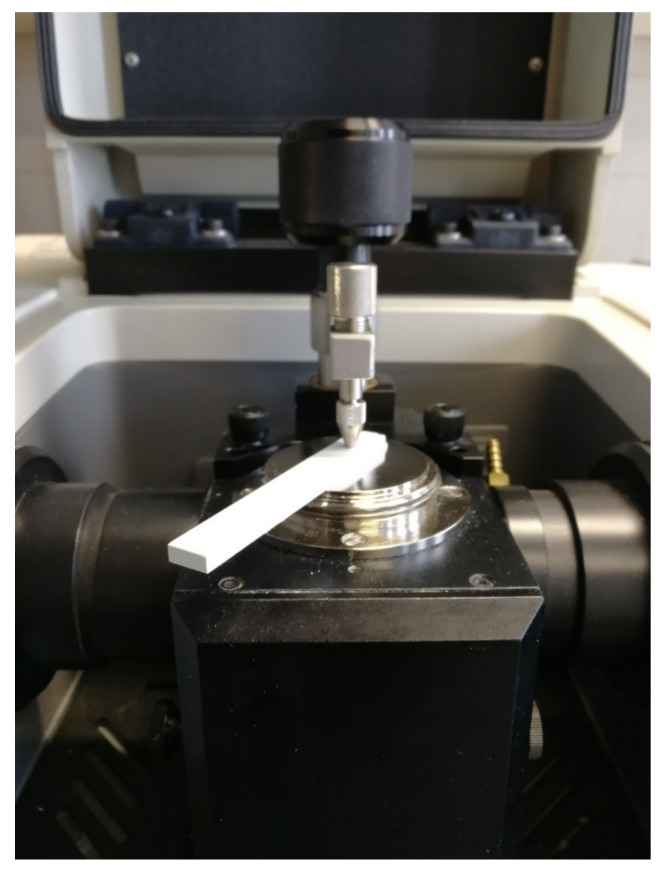
Clamping of the specimen by ATR crystal.

Measurements of the FTIR spectra were done with an AVATAR 320 machine provided by Nicolet (Watertown, MA, USA). OMNIC software (4 cm^−1^ resolution and 64 images) with its reflective method ATR utilizing ZnSe (zinc selenide) crystal was used. To obtain accurate measurements, the FTIR spectrum of the background, in this case air, was scanned at the beginning and at the end of each step (Figure A4). The measurements were done on three specimens for each TAIC concentration and radiation dose.

### 2.7. Atomic Force Microscopy (AFM)

**Figure A5:**
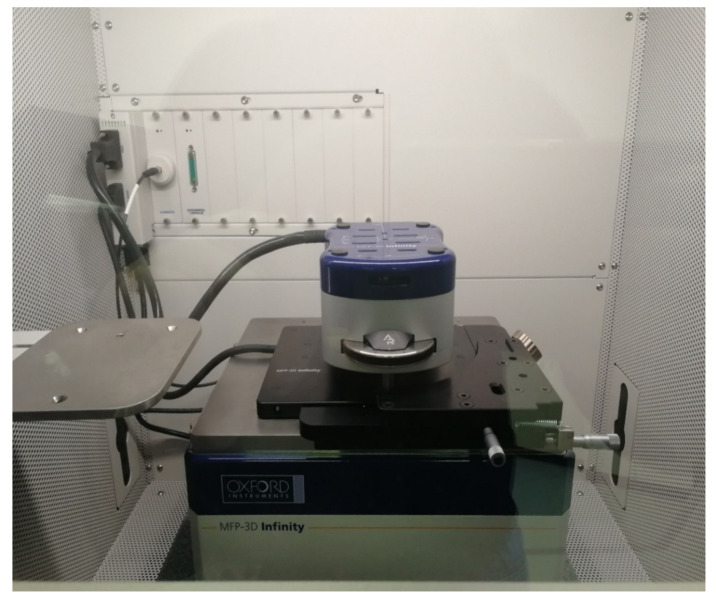
Clamping of the specimen in the device.

Atomic force microscopy was performed with an MFP-3D Infinity device constructed by Oxford Instruments (Abingdon, UK). The measurements were done on three specimens with various TAIC concentrations and radiation doses. The readings were done at approximately the same spot every time, which simplified the evaluation of the data. The silicon probe AC160TS-R3 with rigidity k = 26 N/m was chosen for its visible top and suitability for tapping mode. This probe is suitable for measurements in a frequency range of 200–400 kHz (Figure A5).

## 3. Results

A cross-linking agent is necessary when it comes to polyamides, which undergo only degradation in its absence. As such, it is important to choose the appropriate concentration of the cross-linking agent, as well as the right amounts of radiation. This is quite important, as the correct selection of processing conditions leads to lower cost and thus to higher gains.

### 3.1. Micro-Indentation Properties

The results of the micro-mechanical properties are displayed as median, minimum, and maximum, which are calculated from 10 measurements, which are then compared in dependence on TAIC concentration and radiation dose. The curves shown in graphs represent the trend dependence of indentation hardness on TAIC concentration.

**Figure A6:**
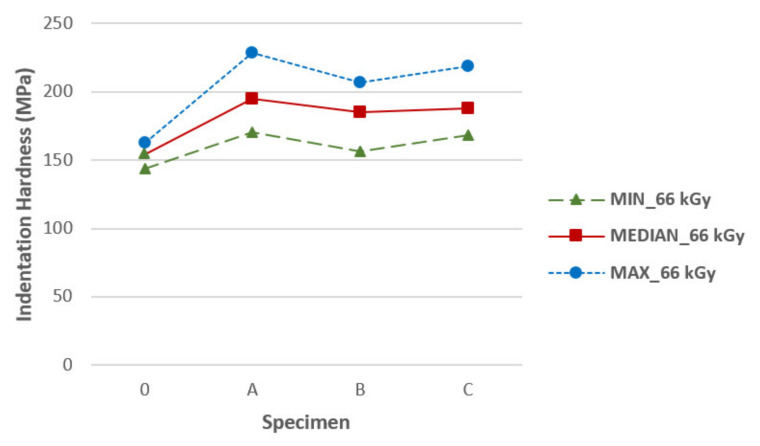
Median, minimum, and maximum indentation hardness in dependence on TAIC concentration (radiation dose 66 kGy).

**Figure A7:**
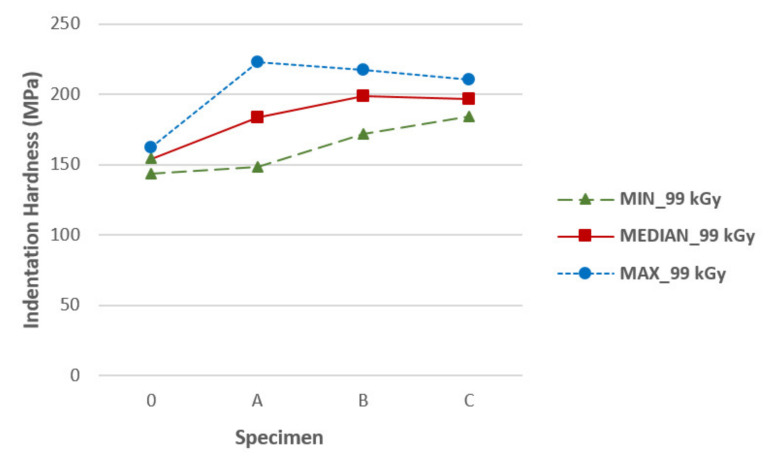
Median, minimum, and maximum indentation hardness in dependence on TAIC concentration (radiation dose 99 kGy).

**Figure A8:**
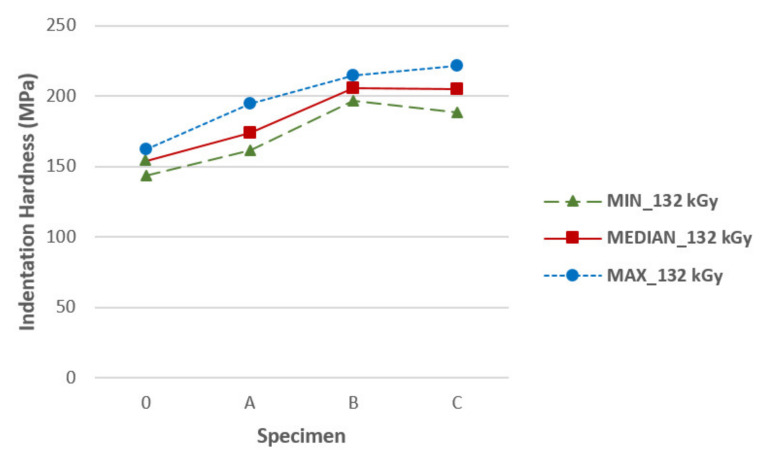
Median, minimum, and maximum indentation hardness in dependence on TAIC concentration (radiation dose 132 kGy).

Figure A6 displays the incremental increase of indentation hardness in dependence on the volume of the cross-linking agent. As is evident from the results, the indentation hardness rose to 190 MPa in specimen A, while it slightly declined in specimens with higher TAIC concentrations. The highest spread of values between the minimum and the maximum indentation hardness was found in specimen A. In case of these specimens, the indentation hardness was in a range of 170 to 223 MPa. This spread was most likely caused by the incomplete cross-linking of the material, which could mean that the radiation dose of 66 kGy was not sufficient for the thorough creation of the 3D network. The application of 99 kGy of radiation led to a continuous increase of indentation hardness with increasing concentration of cross-linking agent TAIC (Figure A7). The highest indentation hardness (200 MPa) was found in specimen B, while the values leveled in specimens with higher concentration. As can be seen from the difference between the minimum and maximum value, the biggest spread of the indentation hardness results was observed in specimen A. The spread range was from 148 to 223 MPa. Higher TAIC concentrations led to a lower spread. The lowest spread range, i.e., 184 to 210 MPa, was found in specimen C. As is evident from Figure A8, the indentation hardness of specimens exposed to 132 kGy of radiation rose, up to test samples with 3 wt.% TAIC concentration. Test subjects with higher TAIC concentration demonstrated a decline in indentation hardness, for which the degradation processes caused by irradiation could be at fault. The spread of values between the minimum and the maximum is quite low for specimens with varying TAIC concentrations. The lowest spread, in a range of 196 to 214 MPa, was found in specimen B.

**Figure 4:**
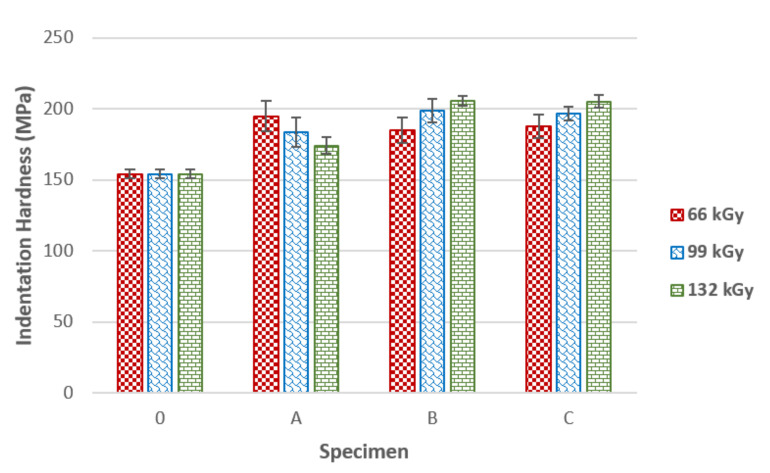
Indentation hardness of irradiated PA 6 in dependence on TAIC concentration.

As can be seen in Figure 4, applying various concentrations of cross-linking agent TAIC influences how good the mechanical properties and the quality of the irradiated product will be. The indentation hardness of the virgin sample, i.e., with no incorporated TAIC, was 154 MPa. The highest increase of indentation hardness in specimen A was found in a sample irradiated by 66 kGy. On the other hand, higher radiation doses led to a decrease of indentation hardness, which could have been caused by the insufficient concentration of the cross-linking agent.

Furthermore, this also influenced the spread of indentation hardness values. Higher radiation doses caused degradation to prevail over cross-linking. Specimens B and C exhibited similar indentation hardness trends as test sample A. The highest indentation hardness (206 MPa) in between specimen B was found in those irradiated by 132 kGy. The difference in indentation hardness between these samples and the virgin material was 34%.

Similar to the previous case, the highest indentation hardness (205 MPa) among specimen C was found in sample irradiated by 132 kGy. The difference between the treated material and the unaltered one was 33%. As the evidence suggests, the optimal radiation dose for both specimens B and C seems to be 132 kGy. Nevertheless, specimen C appears to provide the most equal indentation hardness increase throughout the entire specimen volume, as the spread of values was lowest. These results were confirmed by both the TMA and FTIR measurements.

**Figure 5:**
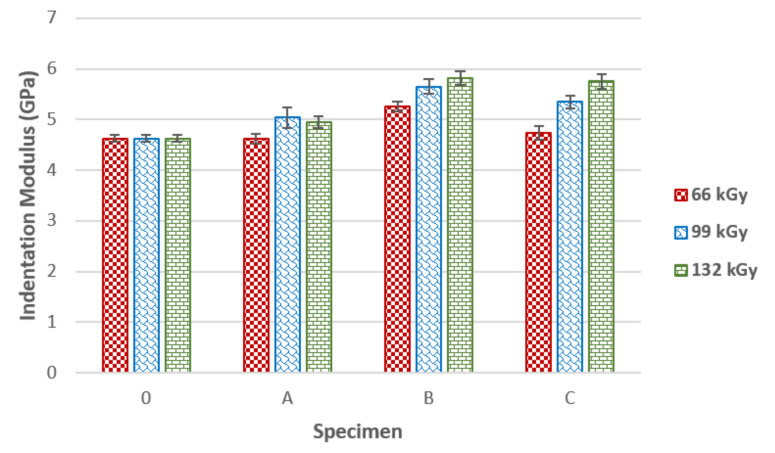
Indentation modulus of irradiated PA 6 in dependence on TAIC concentration.

**Figure A9:**
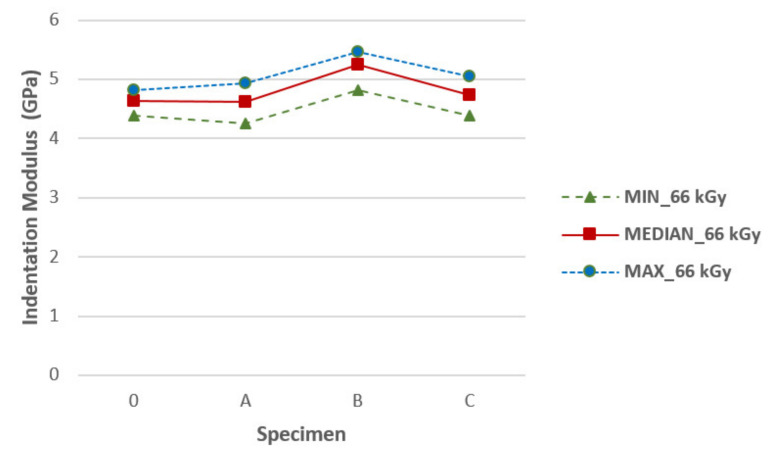
Median, minimum, and maximum indentation modulus in dependence on TAIC concentration (radiation dose 66 kGy).

**Figure A10:**
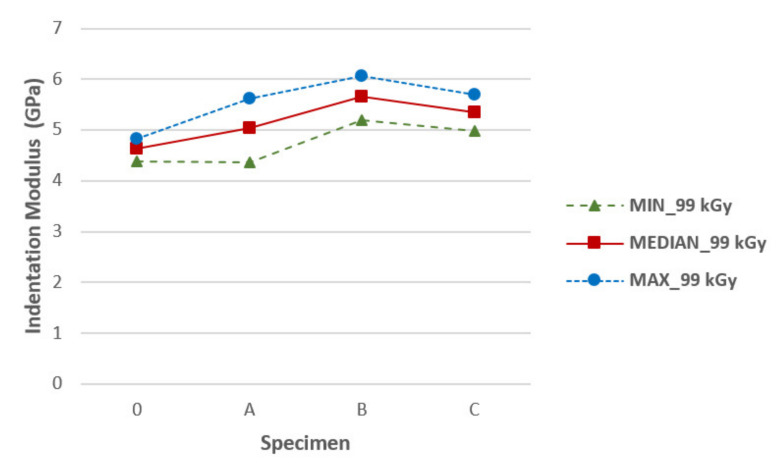
Median, minimum, and maximum indentation modulus in dependence on TAIC concentration (radiation dose 99 kGy).

**Figure A11:**
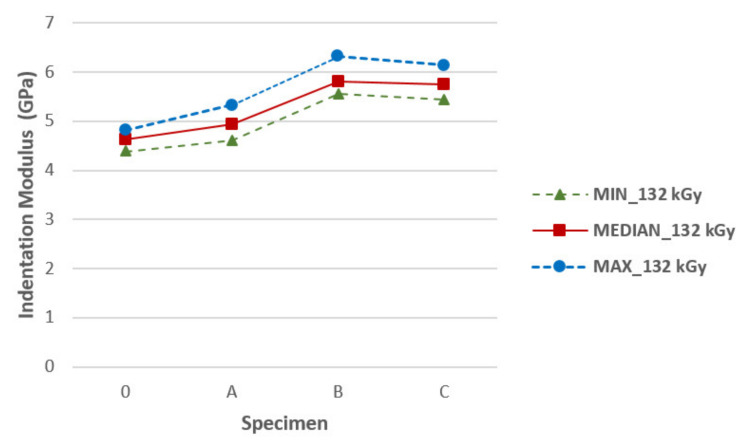
Median, minimum, and maximum indentation modulus in dependence on TAIC concentration (radiation dose 132 kGy).

As can be seen in Figure A9, the exposure of PA 6 to 66 kGy of radiation led to significant variance in the indentation modulus results. Therefore, the aforementioned dose appears to be insufficient in achieving equally cross-linked material. Figure A10 displays the indentation modulus of test subjects irradiated by 99 kGy, which fluctuates between 4.6 and 5.7 GPa. The lowest indentation modulus spread was found in sample C, while the highest spread was found in specimen A. The difference in indentation modulus between the unaltered sample and specimen B was 24%. The ideal TAIC concentration for 132 kGy of radiation appears to be 5 wt.% (specimen C), as can be seen in Figure A11. The values of indentation modulus in this case can be found in a range from 5.3 to 6.1 GPa. PA 6 modified in such a way can be equally cross-linked by a 132 kGy radiation dose. According to median of the indentation modulus, the 132 kGy radiation appears to provide the best results and spread of values for all TAIC concentrations. The highest values of indentation modulus (5.8 GPa) were found in specimen C exposed to 132 kGy of radiation. These measurements once again show that PA 6 demonstrates the best results when exposed to 132 kGy of radiation. The increase of indentation hardness in this sample was 26% in comparison with the untreated sample. As shown in Figure 5, a suitable radiation dose and TAIC concentration leads to significant improvement in indentation modulus. Unaltered material that was not irradiated was measured to have a 4.6 GPa indentation modulus. A radiation dose of 66 kGy was not sufficient for any significant improvements in indentation modulus. Only a slight increase of modulus was found in sample B, while the irradiated specimen C demonstrated values similar to untreated material.

Specimen A was not cross-linked, since the degradation processes were prevalent. The indentation modulus rose significantly in specimens irradiated by 99 and 132 kGy of radiation. Specimen B had the highest indentation modulus (5.7 GPa) when irradiated by 99 kGy, while test sample C had the best results (5.8 GPa) after exposure to 132 kGy of radiation. The most suitable combination appears to be 5 wt.% of TAIC and 132 kGy of radiation. The difference between the untreated PA 6 and PA 6 with 5 wt.% of TAIC exposed to 132 kGy of radiation was 26%.

### 3.2. Thermo-Mechanical Analysis (TMA)

**Figure 6:**
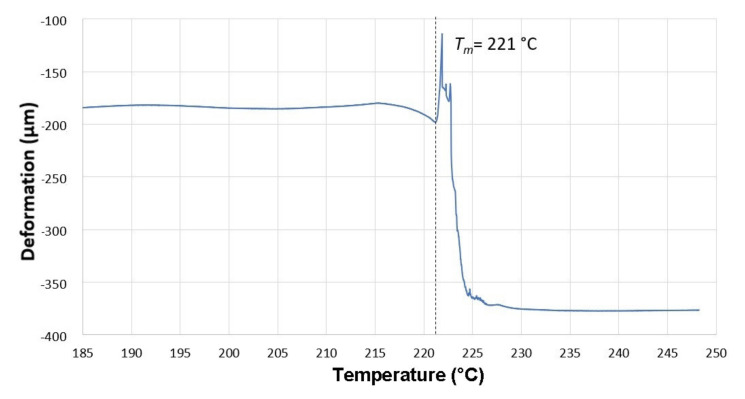
Influence of temperature on the deformation of non-irradiated test samples (0 kGy).

Thermo-mechanical analysis was used to measure two processes, i.e., thermal expansion and the flow of the non-cross-linked part. These two processes influence each other, and the dominating one has a direct effect on whether the curve grows due to thermal expansion or declines due to thermal deformation. The curves in the following graphs consist of medians of three measurements. For a better visualization of the measured trend, the material deformation was taken at three temperatures: 230, 240, and 248 °C (Figure 6).

**Figure 7:**
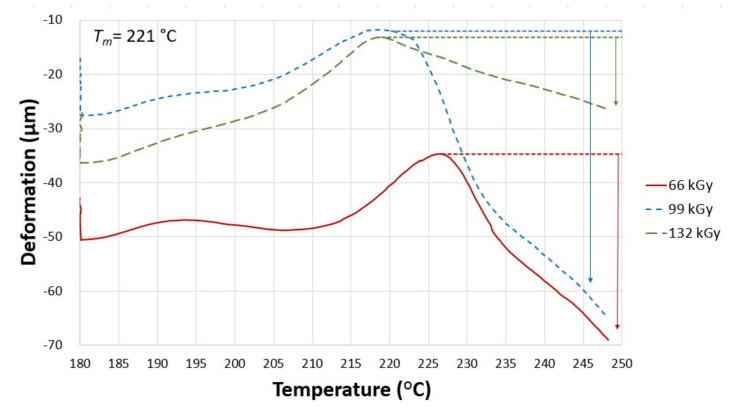
Influence of temperature on the deformation of specimen A (1 wt.% of TAIC) irradiated by 66, 99, 132 kGy.

As shown in Figure 7, specimen A exposed to 66 kGy of radiation has different mechanical behavior than untreated PA 6. The dotted line represents a tangent, from which the deformation for 3 temperatures (230, 240, and 248 °C) was taken. The thermal expansion of the solid, partly crystalline phase occurs in a temperature range of 185 °C to 192 °C, while the deformation of the test sample happens in a temperature range of 192 °C to 207 °C, which is once again followed by the thermal expansion of the solid phase. Starting from 227 °C, the specimen undergoes rapid deformation, which lasts up until 248 °C. The subsequent deformations were measured at three points: −0.13% at 230 °C, −0.59% at 240 °C, and −0.86% at 248 °C.

Figure 7 also displays the TMA curve of specimen A, which was irradiated by 99 kGy. The trend is similar to the previous sample. The following deformations were measured at the designated points: −0.64% at 230 °C, −1.04% at 240 °C, and −1.32% at 248 °C.

Finally, Figure 7 shows the TMA curve of test sample A, which was irradiated by the highest used dose, 132 kGy. According to the result, this specimen demonstrated the lowest deformations in between the samples with the given TAIC concentration (1 wt.%). The following deformations were measured: −0.14% at 230 °C, −0.24% at 240 °C, and −0.33% at 248 °C. From a mechanical behavior point of view, it can be said that this radiation dose led to improved cross-linking in comparison with the previous specimen.

**Figure 8:**
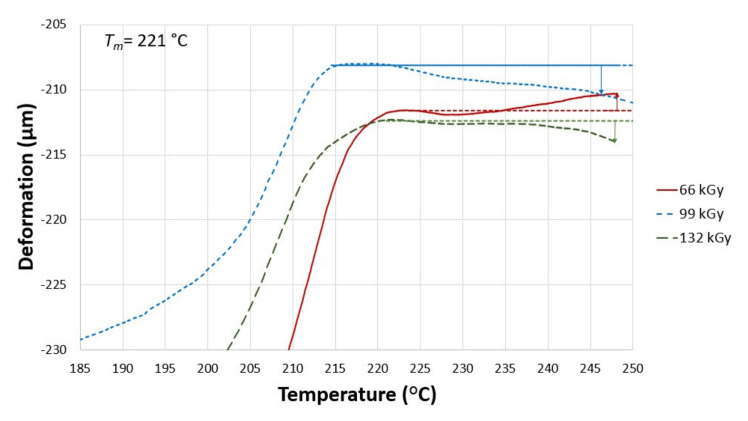
Influence of temperature on the deformation of specimen B (3 wt.% of TAIC), which was irradiated by 66, 99, 132 kGy.

Specimen B (Figure 8), specifically the test sample irradiated by 66 kGy, demonstrated domination by thermal expansion in the range 230 °C to 248 °C. The deformation of the test subject taken at three various points was the following: 0% at 230 °C and 240 °C and 0.03% at 248 °C. These results imply that increasing the TAIC concentration could lead to improved cross-linking and consequently, a rising TMA curve.

The deformation of specimen B, which was irradiated by 99 kGy, was once again almost inconsequential, i.e., −0.02% at 230 °C, −0.05% at 240 °C, and −0.06% at 248 °C.

A similar trend was observed in the same sample that was exposed to 132 kGy of radiation. The test sample exhibited the following deformations: 0% at 230 °C, −0.01% at 240 °C, and −0.04% at 248 °C. Such results imply that the material is sufficiently cross-linked.

**Figure 9:**
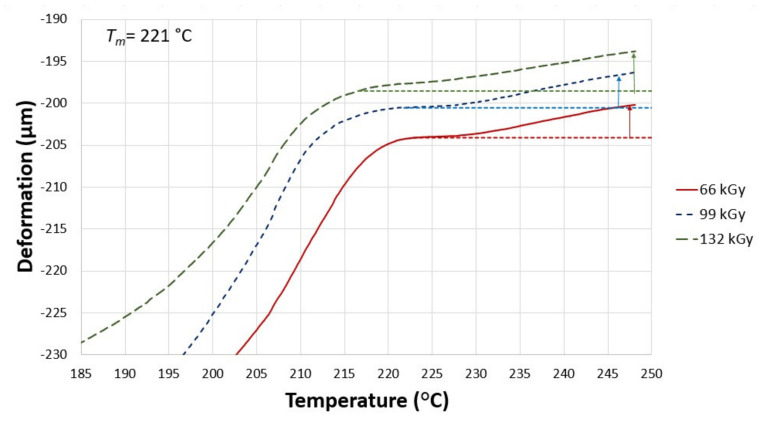
Influence of temperature on the deformation of the test sample C (5 wt.% of TAIC) that was irradiated by 66, 99, 132 kGy.

A similar trend as with specimen B irradiated by 132 kGy was found in specimen C irradiated by 66 kGy (Figure 9). The thermal expansion was 0% at 230 °C, 0.06% at 240 °C, and 0.1% at 248 °C. There was no deformation in this case. Higher thermal expansion was noted in sample C that was exposed to 99 kGy of radiation. This can be seen in Figure 9, which shows the following thermal expansion: 0.02% at 230 °C, 0.07% at 240 °C, and 0.11% at 248 °C. The highest thermal expansion in this bracket was found in specimen C, which was irradiated by 132 kGy. The thermal expansion was 0.04% at 230 °C, 0.08% at 240 °C, and 0.12% at 248 °C.

**Figure 10:**
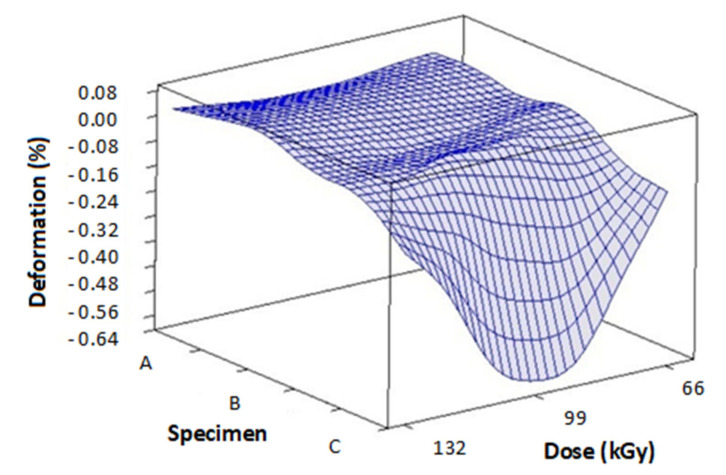
Influence of radiation dose and TAIC concentration on the deformation of specimens measured at 230 °C.

**Figure 11:**
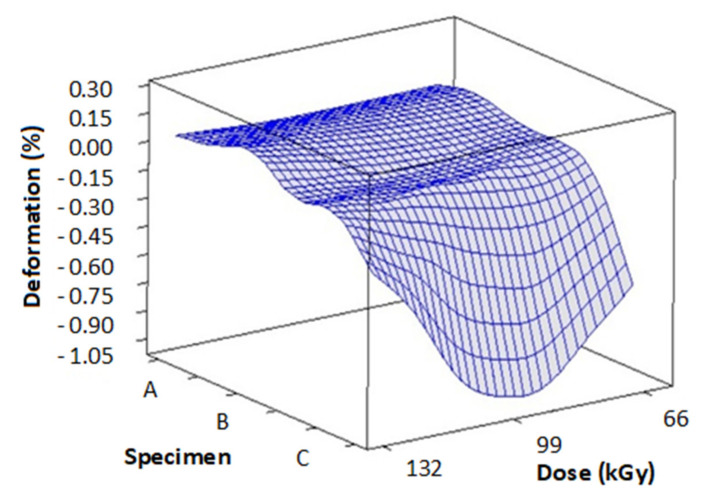
Influence of radiation dose and TAIC concentration on the deformation of specimens (measured at 240 °C).

**Figure 12:**
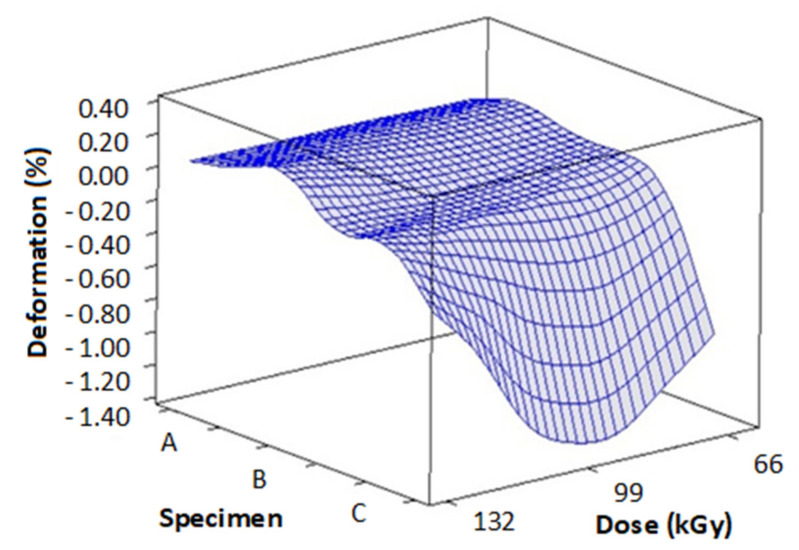
Influence of radiation dose and TAIC concentration on specimens’ deformation (measured at 248 °C).

Figure 10 and Table 4 contains the measurements of deformation and thermal expansion in dependence on TAIC concentration and radiation dose. These readings were done at 230 °C. Figure 11 and Table 5 displays the results of deformation and thermal expansion in dependence on TAIC concentration and radiation dose. These measurements were taken at 240 °C. Figure 12 and Table 6 shows the measurements of deformation and thermal expansion in dependence on TAIC concentration and radiation dose. These readings were done at 248 °C. The results imply that the most suitable concentration/radiation combination for specimen C and 132 kGy of radiation. This combination caused the biggest improvements in mechanical properties above the melt temperature.

The deformation measurements made at 230 °C (Figure 10) show that sample A irradiated by 99 kGy had the worst deformation (−25.46 µm) and thermal expansion (−0.64 µm), which indicates insufficient cross-linking and prevailing degradation. Similar results could be observed in the same specimen irradiated by 132 kGy and in sample B, which was exposed to 99 kGy of radiation. On the other hand, the best values of deformation (1.7 µm) and thermal expansion (0.04 µm) were found in specimen C irradiated by 132 kGy and 99 kGy (0.7 µm deformation, 0.02 µm). These results indicate significant prevalence of cross-linking over degradation.

Deformation measurements performed at 240 (Figure 11) and 248 °C (Figure 12) unraveled similar tendencies as in measurements made at 230 °C. Just like with the previously measured temperature, the worst deformation at 240 °C (−41.65 µm) and 248 °C (−52.94 µm) was found in sample A irradiated by 99 kGy. These results could be explained by insufficient amounts of cross-linking agent being present in PA 6. On the contrary, specimen C irradiated by 99 and 132 kGy contained the ideal mass of cross-linking agent, which manifested in great deformation values. These results were subsequently confirmed by the measurements of hardness and modulus, in which these specimens also demonstrated the best values.

### 3.3. Fourier-Transform Infrared Spectroscopy (FTIR)

**Figure 13:**
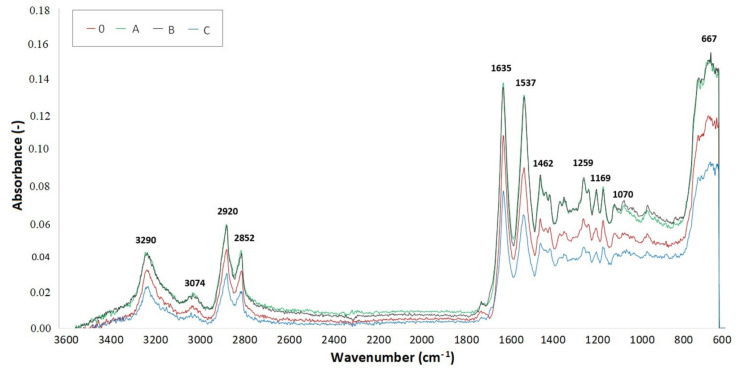
FTIR spectrum of PA 6 irradiated by 66 kGy.

**Figure 14:**
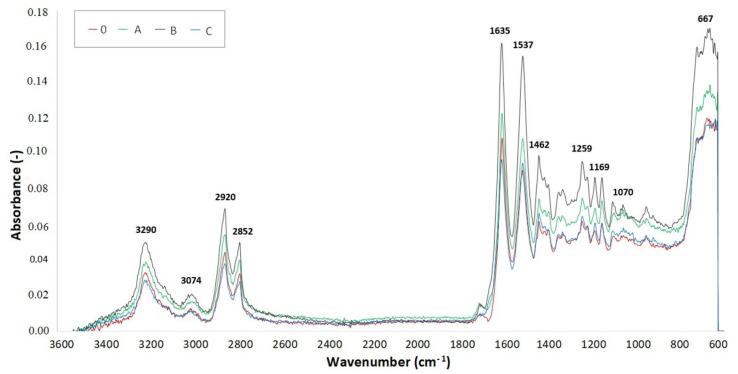
FTIR spectrum of PA 6 irradiated by 99 kGy.

**Figure 15:**
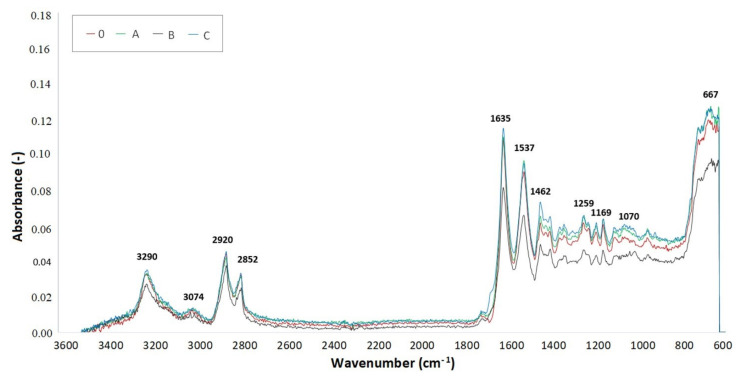
FTIR spectrum of PA 6 irradiated by 132 kGy.

The spectrum curves were created out of the medians of three measurements for each test sample. Infrared spectroscopy was used to determine the presence of individual chemical groups. Figure 13, Figure 14 and Figure 15 display FTIR for test samples irradiated by 66, 99, and 132 kGy. Comparison of the IR spectra proved minor material degradation, which is given by the 1736 cm^−1^ region that is typical for carbonyl groups.

Furthermore, the presence of hydroxyl groups, which belong within region 3500 cm^−1^, was not found in measured specimens. Vibrations at 3290, 3074, 1635, 1537, 964, and 667 cm^−1^ prove the presence of amide group. On the other hand, vibrations at 2920, 2852, 1462, 1352, and 1259 cm^−1^ signify the presence of a CH_2_ group, thus confirming the presence of an aliphatic chain. The intensity of these regions declines with increasing radiation dose, which could be caused by the reduced mobility of the CH_2_ group.

Crystalline phase α is represented by a vibration with a wavenumber of 1201 cm^−1^, which comes from the fanning vibration of CH_2_ group. The width of this region increases in irradiated specimens. The region with a wavenumber of 1697 cm^−1^, which is associated with cross-linking accelerator TAIC, was not found in the FTIR spectrum of any examined sample. This could mean that the cross-linking agent was consumed during the radiation-induced processes.

The lowest location of peaks for radiation doses 66 and 99 kGy were found in sample C due to significant cross-linking. In comparison, the baseline was moved upwards in graphs describing samples A and B, which means the cross-linking processes were not that significant. These results are supported by the micro-mechanical properties measurements. Furthermore, out of all samples irradiated by 132 kGy of radiation, it was specimen B that displayed the greatest hardness, which corresponds with its lowest peak position in Figure 15.

Table 7 displays the values of absorbance around wavenumber 1736 cm^−1^, which is typical in the presence of carbonyl groups signifying the degradation process of PA 6. The higher the absorbance value, the greater the radiation-induced degradation of the material. On the other hand, the lowest values of absorbance indicate a significant degree of cross-linking. The lowest values of absorbance in sample C were found in specimens irradiated by 99 kGy. The lowest absorbance (0.00988) is highlighted by the green color. This absorbance corresponds with the measurements of micro-mechanical properties, in which the specimen C irradiated by 99 kGy had the best indentation hardness and modulus. Concurrently, the TMA analysis confirmed that this sample was the most cross-linked specimen out of all tested samples. In sample C irradiated by 132 kGy, the degradation prevailed over cross-linking due to high amounts of radiation, which resulted in an increase of absorbance.

The highest values of absorbance for samples A and B were found in those that were irradiated by 66 and 99 kGy, which indicates an imperfect cross-linking process. The red highlighted absorbance value (0.01552) belongs to sample A irradiated by 66 kGy. On the contrary, the lowest values of absorbance were measured in samples irradiated by 132 kGy, which means the material underwent cross-linking.

The cross-linking of polymers is a chemical process in which transverse bonds are created within the structure of the polymer. The transverse bonds enable the creation of an infinite 3D structure, i.e., a spatial network or gel. This process encompasses two contradictory events, i.e., cross-linking and scission, which happen at the same time. The irradiation of polyamides in the presence of oxygen (a typical interceptor of radicals) leads to not only cross-linking, but also fragmentation, disproportionation, or oxidation, which manifest by chain degradation, which could lead to brittle material.

The measurements of mechanical properties, TMA and FTIR show that the optimal amount of cross-linking agent and radiation dosage is pivotal for the improvement of these properties. If the cross-linking agent content is insufficient (1 and 3 wt.%), then degradation prevails over cross-linking. This effect can be observed in specimens irradiated by 66 and 99 kGy. Results show that the optimal amounts of TAIC was 5 wt.%, which enables cross-linking even for lower radiation dosages. Once the radiation dosage reaches 132 kGy, then degradation starts to prevail over cross-linking.

### 3.4. Formatting of Mathematical Components

In order to map the topography of the specimens, atomic force microscopy was used. The results that were gained could not be used to conclusively determine the influence of irradiation on the topography of PA 6.

**Figure 16:**
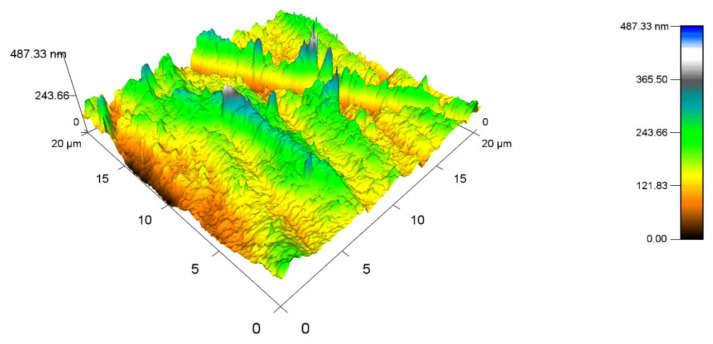
Surface topography of the virgin test sample (0 kGy).

**Figure 17:**
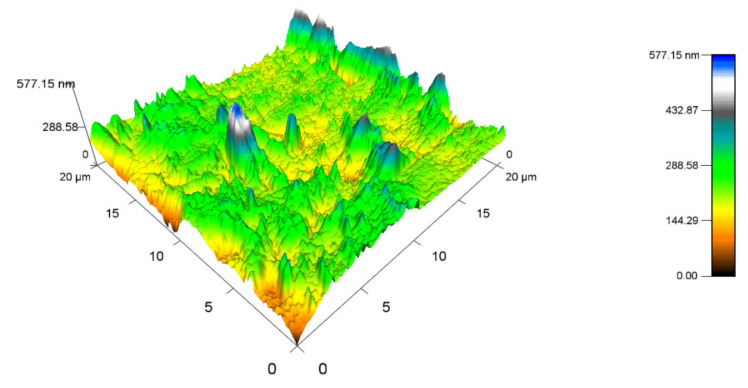
Surface topography of test sample A irradiated by 132 kGy.

**Figure 18:**
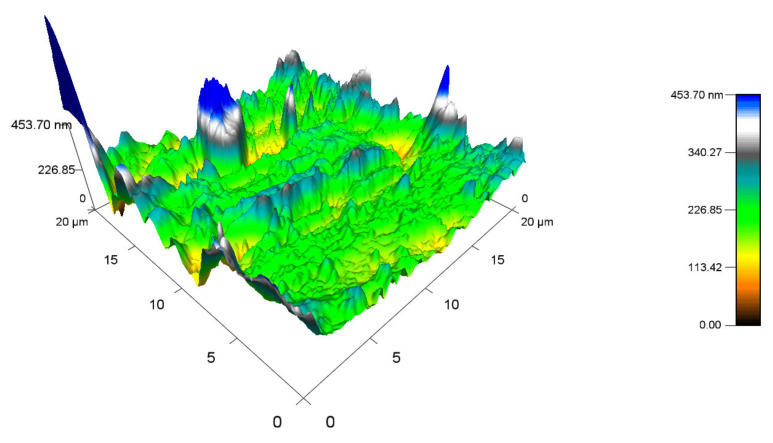
Surface topography of test sample B irradiated by 132 kGy.

**Figure 19:**
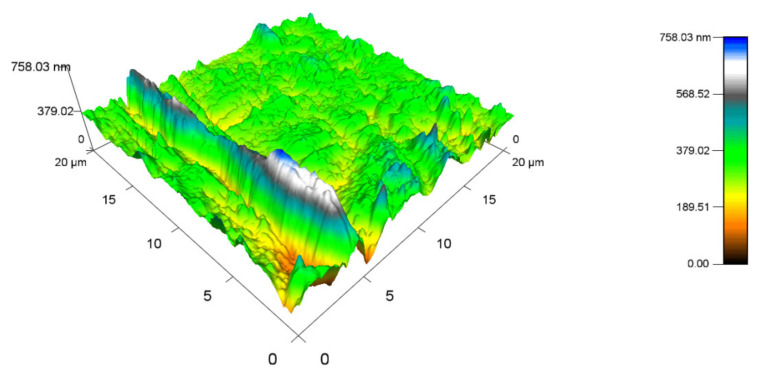
Surface topography of test sample C irradiated by 132 kGy.

Figure 16 displays the topography of the PA 6 surface that was not exposed to any radiation. The highest peak measured 487.33 nm (Ra 0.8), while the minimum was close to 0 nm. The topography of sample A, which was irradiated by 132 kGy (Figure 17), was measured to have the highest peak, at 577.15 nm (Ra 0.9), which is similar to virgin PA 6. The highest peak of test sample B (Figure 18) was 453.7 nm (Ra 0.8), which is once again like unaltered PA 6. Specimen C (Figure 19) had the highest peak, at 758.03 nm (Ra 1).

The topography measurements investigated whether the surface quality was affected by electron radiation in a range of 66 to 132 kGy. No significant change in the topography of individual samples was found; thus it can be said that the application of electron beam does not change the topography of the surface. This is an especially important finding for micro-mechanical measurement, as any quality decline of the surface can worsen the accuracy of the results.

## 4. Conclusions

This study investigated the influence of cross-linking accelerator (TAIC) concentration on the final micro-mechanical properties of PA 6 irradiated by 66, 99, and 132 kGy. As the evidence of the specimens’ cross-linking, TMA analysis concerned with thermal deformation and expansion was performed. Furthermore, the effect of degradation was investigated by FTIR spectroscopy, while the surface topography was observed by AFM microscopy.

The results have shown that, to obtain maximum cross-linking, minimum degradation, and the highest increase of indentation hardness and modulus, PA 6 had to contain either 3 or 5 wt.% of TAIC and be irradiated by 132 kGy. This combination proved to be most suitable according to the results discussed above.

The TMA findings proved that specimen with 5 wt.% of TAIC irradiated by 132 kGy was the most cross-linked sample. This sample experienced thermal expansion above 220 °C in the following manner: 0.04% at 230 °C, 0.08% at 240 °C, and 0.12% at 248 °C. There was no recorded degradation in this material. These values of thermal expansion could mean that the material was sufficiently cross-linked; thus, it is not necessary to increase the TAIC concentration or the radiation dose.

The values of absorbance, with a wavenumber of 1736 cm^−1^, which is typical for carbonyl groups, indicate the degradation of PA 6. The lowest absorbance was 0.00613, which was found in PA 6 with 5 wt.% of TAIC irradiated by 132 kGy. This corresponds with the aforementioned results. On the other hand, absorbance 0.01552 was found in a specimen with 1 wt.% of TAIC irradiated by 99 kGy, which demonstrated the highest degradation in this research.

The surface topography was mapped to provide an overview of a possible effect of ionizing radiation on the PA 6 surface. The AFM mapping shows that applied doses have no influence on the topography of the PA 6 surface, which means that there should be no possibility of changes to the surface topography influencing the final properties of the material when measured.

Nowadays, metal materials in industrial sectors are often replaced by plastic and composite parts. Therefore, it is important to find a suitable method of material modification, which is environmentally friendly and cheap, while positively influencing the material. Each application requires different mechanical, thermal, and chemical properties. Although material sheets contain information regarding the material properties, the surface layer properties are not a part of these sheets.

## Figures and Tables

**Table 1:** Alternative label for tested specimens.

Specimen	Polymer	TAIC Concentration (wt.%)
0	Polyamide 6	0
A	Polyamide 6	1
B	Polyamide 6	3
C	Polyamide 6	5

**Table 2:** Technological parameters settings for individual materials.

Technological Parameter	Unit	Value
Injection pressure	MPa	80
Injection velocity	mm/s	50
Length of a dose	mm	26
Temperature under the hopper	°C	70
Cooling time	s	20
Holding pressure	MPa	68
Holding pressure duration	s	10
Heat zones settings		
Zone n. 1	°C	220
Zone n. 2	°C	230
Zone n. 3	°C	245
Zone n. 4	°C	265

**Table 3:** Process parameters of TMA.

Technological Parameter	Unit	Value
Initial temperature	°C	180
Temperature-stabilization duration	min	10
Load	N	1
Heating velocity	K/min	1
Final temperature	°C	250

**Table 4:** Deformation results of test samples measured at 230 °C.

Radiation Dose (kGy)	Specimen	Deformation [Specimen Deformation (−) + Thermal Expansion (+)] (%)	Deformation (µm)
66	A	−0.13	−5.33
99	A	−0.64	−25.46
132	A	−0.14	−5.75
66	B	0.00	0.00
99	B	−0.02	−0.88
132	B	0.00	0.00
66	C	0.00	0.00
99	C	0.02	0.70
132	C	0.04	1.70

**Table 5:** Deformation results of specimens measured at 240 °C.

Radiation Dose (kGy)	Specimen	Deformation [Specimen Deformation (−) + Thermal Expansion (+)] (%)	Deformation (µm)
66	A	−0.59	−23.74
99	A	−1.04	−41.65
132	A	−0.24	−9.75
66	B	0.00	0.00
99	B	−0.05	−1.88
132	B	−0.01	−0.42
66	C	0.06	2.43
99	C	0.07	2.71
132	C	0.08	3.37

**Table 6:** Deformation results of the test samples measured at 248 °C.

Radiation Dose (kGy)	Specimen	Deformation [Specimen Deformation (−) + Thermal Expansion (+)] (%)	Deformation (µm)
66	A	−0.86	−34.01
99	A	−1.32	−52.94
132	A	−0.33	−13.33
66	B	0.03	1.32
99	B	−0.06	−3.00
132	B	−0.04	−1.65
66	C	0.10	3.92
99	C	0.11	4.24
132	C	0.12	4.74

**Table 7:** Absorbance value at wavenumber 1736 cm^−1^.

Specimen	Absorbance
0% TAIC, 0 kGy	0.00979
A, 66 kGy	0.01805
A, 99 kGy	0.01552
A, 132 kGy	0.01097
B, 66 kGy	0.01519
B, 99 kGy	0.01529
B, 132 kGy	0.01151
C, 66 kGy	0.01519
C, 99 kGy	0.00988
C, 132 kGy	0.01225

## Data Availability

The data presented in this study are available on request from the corresponding author.

## References

[1] Drobny J.G. (2010). Radiation Technology for Polymers.

[2] Mark J.E. (2007). Physical Properties of Polymers Handbook.

[3] Dadbin S., Frounchi M., Goudarzi D. (2005). Electron beam induced crosslinking of nylon 6 with and without the presence of TAC. Polym. Degrad. Stab..

[4] Pramanik N.K., Haldar R.S., Bhardwaj Y.K., Sabharwal S., Niyogi U.K., Khandal R.K. (2009). Radiation processing of Nylon 6 by e-beam for improved properties. Radiat. Phys. Chem..

[5] Holík Z., Daněk M., Maňas M., Černý J. (2011). Influence of the Amount of Cross-linking Agent on Properties of Irradiated Polyamide 6. Int. J. Mech..

[6] Ovsik M., Manas D., Manas M., Stanek M., Sanda S., Kyas K., Reznicek M. (2012). Microhardness of PA6 Influenced by Beta Low Irradiation Doses. Int. J. Math. Comput. Simul..

[7] Porubska M., Janigova I., Jomova K., Hodak I.C. (2014). The effect of electron beam irradiation on properties of virgin and glass fiber-reinforced polyamide 6. Radiat. Phys. Chem..

[8] Adem E., Burillo G., del Castillo L.F., Vasquez M., Avalos-Borja M., Marcos-Fernandez A. (2014). Polyamide-6: The effects on mechanical and physiochemical properties by electron beam irradiation at different temperatures. Radiat. Phys. Chem..

[9] Manas D., Ovsik M., Manas M., Stanek M., Spanhelova M., Bednarik M., Senkerik V. (2015). Influence of Content of Crosslinking Agent on the Micromechanical Properties of Glass-Filled Polyamide 6. Appl. Mech. Mater..

[10] Shin B.Y., Ha M.H., Han D.H. (2016). Morphological, Rheological, and Mechanical Properties of Polyamide 6/Polypropylene Blends Compatibilized by Electron-Beam Irradiation in the Presence of a Reactive Agent. Materials.

[11] Manas D., Ovsik M., Mizera A., Manas M., Hylova L., Bednarik M., Stanek M. (2018). The Effect of Irradiation on Mechanical and Thermal Properteis of Selected types of Polymers. Polymers.

[12] Bradler P.R., Fischer J., Wallner G.M., Lang R.W. (2019). Characterization of irradiation crosslinked polyamides for solar thermal applications—Fatigue properties. Compos. Sci. Technol..

[13] Yang Y., Xi C., Lu N., Gao F. (2016). Injection Molding Process Control, Monitoring and Optimization.

[14] Makuuchi K., Cheng S. (2012). Radiation Processing of Polymer Materials and Its Industrial Applications.

[15] Porubska M., Babic D., Janigova I., Slouf M., Klaudia J., Chodak I. (2016). The effect of gamma irradiation in air and inert atmosphere on structure and properties of unfilled or glass fibre-reinforced polyamide 6. Polym. Bull..

[16] Beaumont J.P., Nagel R.L., Sherman R. (2002). Successful Injection Molding: Process, Design, and Simulation.

[17] Ashcroft W.R. (2017). Industrial Polymer Applications: Essential Chemistry and Technology.

[18] Ferreira T., Lopes P.E., Pontes A.J., Paiva M.C. (2016). Microinjection molding of polyamide 6. Polym. Adv. Technol..

[19] Oliver W.C., Pharr G.M. (2004). Measurement of hardness and elastic modulus by instrumented indentation: Advances in understanding and refinements to methodology. J. Mater. Res..

[20] Manas D., Mizera A., Navratil J., Manas M., Ovsik M., Sehnalek S., Stoklasek P. (2018). The electrical, mechanical and surface properties of thermoplastic polyester elastomer modified by electron beta radiation. Polymers.

[21] Manas D., Mizera A., Manas M., Ovsik M., Hylova L., Sehnalek S., Stoklasek P. (2018). Mechanical properties changes of irradiated thermoplastic elastomer. Polymers.

[22] Ovsik M., Manas M., Stanek M., Dockal A., Vanek J., Mizera A., Adamek M., Stoklasek P. (2020). Polyamide surface layer nano-indentation and thermal properties modified by irradiation. Materials.

